# Study protocol: A randomized controlled trial of a community-based intervention on clinical parameters, health literacy, medication adherence, and quality of life among individuals with coexisting diabetes and hypertension

**DOI:** 10.1016/j.mex.2025.103700

**Published:** 2025-10-29

**Authors:** Krishna Kumari Samantaray, Sasmita Das, Krushna Chandra Sahoo, N Siva

**Affiliations:** a Department of Community Health Nursing, SUM Nursing College, Shiksha ‘O’ Anusandhan (SOA) University, Bhubaneshwar, Odisha, India; b Department of Medical and Surgical Nursing, SUM Nursing College, Shiksha ‘O’ Anusandhan (SOA) University, Bhubaneshwar, Odisha, India; c Health Technology Assessment in India (HTAIn), Department of Health Research, Ministry of Health & Family Welfare, Govt. of India, India; d Department of Child Health Nursing, SUM Nursing College, Siksha ‘O’ Anusandhan (SOA) University, Bhubaneswar, Odisha, India

**Keywords:** Community-based intervention, Diabetes, Hypertension, Medication adherence, Health Literacy, Quality of Life

## Abstract

**Background:**

Diabetes and hypertension are prevalent chronic conditions that often coexist and contribute significantly to morbidity and mortality. Effective management requires patient-centred interventions that integrate lifestyle modification, health education, and self-monitoring.

**Objective:**

This study aims to evaluate the effectiveness of Community-Based Intervention (CBI) on clinical parameters, health literacy, medication adherence, and quality of life among Individuals with coexisting diabetes and hypertension in urban slum settings.

**Methods:**

A randomized controlled trial (RCT) will be conducted among 120 eligible participants aged between 30–60 years. Participants will be randomly assigned to either the intervention or control group using block randomization (12 blocks, each containing 5 participants per group) with allocation concealment. The intervention group will receive a 6-week CBI consisting of pre-intervention orientation, yoga-based lifestyle modification, nurse-led counseling, and home-based monitoring, in addition to standard care. The control group will receive standard care, with informational materials provided after the intervention period.

**Expected Outcomes:**

Data on blood sugar, blood pressure, health literacy, medication adherence, and quality of life will be collected at baseline, 2 months, 3 months, and 6 months. The intervention is expected to improve clinical outcomes, enhance health literacy, increase medication adherence, and promote better quality of life.

## Abstract (author highlights)

• **Ms Krishna Kumari Samantaray:**
• **Dr Sasmita Das and Dr Krushna Chandra Sahoo:**
• **Dr N. Siva:**

## Graphical abstract

**Figure:**
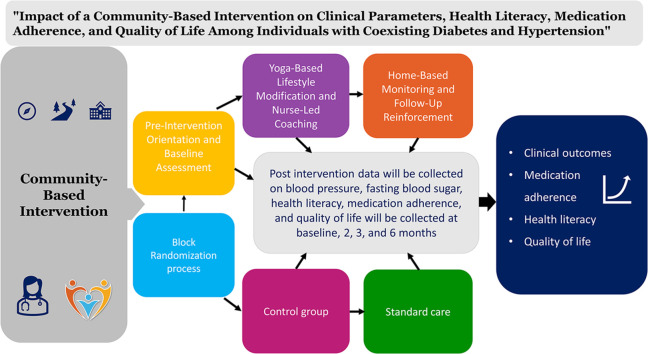

## Specifications table

**Table:** 

**Subject area**	Nursing and Medicine
**More specific subject area**	Community-based care for chronic diseases (Diabetes and Hypertension)
Name of protocol	Community-Based intervention (CBI) for Individuals with Coexisting Diabetes and Hypertension
**Reagents/tools / Instruments**	- Sociodemographic proforma and Clinical Profile Form - Digital Sphygmomanometer (BP measurement) - Standardized Glucometer (Random Blood Sugar Test) - Modified Diabetes and Hypertension Health Literacy Scale - 8-item Morisky Medication Adherence Scale (MMAS-8) - Modified Quality of Life Questionnaire
**Experimental design**	Randomized controlled trials (block randomization) with 1:1 allocation between intervention and control groups. **Intervention group:** Participants will receive the CBI, including: [1] pre-intervention orientation and baseline assessment, [2] yoga-based lifestyle modification and nurse-led counseling (6 days/week for 6 weeks), and [3] home-based monitoring and follow-up reinforcement. **Control group:** Participants will continue standard care and receive informational booklets on yoga and dietary management after intervention completion. Follow-up assessments will be conducted at 2 months, 3 months, and 6 months.
Trail registration	Registered in Clinical Trial Registry of India (CTRI/2024/08/071,775)
Ethics	Approved by Institutional Ethics Committee (IEC) of Institute of Medical Sciences and SUM Hospital, Siksha ‘O’ Anusandhan, Bhubaneswar, Odisha, India (Reference no: IEC/IMS.SH/SOA/2022/433). The study will adhere to the Declaration of Helsinki, ensuring voluntary participation, confidentiality, and participant safety.
Value of the protocol	1) Provides evidence on the effectiveness of a structured community-based intervention for patients with coexisting diabetes and hypertension. 2) Facilitates patient-centered care by integrating yoga, education, and home monitoring into routine treatment. 3) Offers insights for developing scalable interventions in urban slum populations to improve medication adherence, clinical outcomes, and quality of life.

## Background

Diabetes and hypertension are two of the most prevalent noncommunicable diseases (NCDs) worldwide and are leading contributors to morbidity and mortality [1,2]. Diabetes is characterized by persistent hyperglycemia resulting from insufficient insulin secretion or impaired insulin utilization, which over time causes damage to multiple organ systems [3,4]. Hypertension, defined as systolic blood pressure ≥140 mmHg or diastolic blood pressure ≥90 mmHg or current use of antihypertensive medication, increases the risk of cardiovascular disease, stroke, kidney failure, and premature mortality [5]. Globally, the burden of these conditions is rapidly escalating, with more than 529 million people living with diabetes in 2021 a figure projected to reach 700 million by 2045 and nearly one billion people affected by hypertension, expected to rise to 1.56 billion by 2025 [6,7]. Recognising this challenge, the World Health Organisation’s Global Action Plan for the Prevention and Control of NCDs has set targets to halt the rise of diabetes and reduce the prevalence of hypertension, while the United Nations’ Sustainable Development Goal 3 aims to reduce premature mortality from NCDs by one-third by 2030 [8].

India carries a disproportionate share of this global burden, with nearly 69.1 million people living with diabetes and hypertension projected to increase by 80 % between 2000 and 2025 [6]. Alarmingly, hypertension is increasingly common among young adults, raising the urgency for preventive interventions. The coexistence of diabetes and hypertension further complicates disease management, as more than 80 % of patients with type 2 diabetes develop hypertension, and approximately one-fifth of hypertensive patients later develop diabetes [9]. Their shared pathophysiological mechanisms, including insulin resistance, endothelial dysfunction, and obesity, amplify the risk of complications such as atherosclerosis, nephropathy, retinopathy, and cardiovascular disease, ultimately leading to high healthcare costs and adverse socioeconomic impacts [10].

Addressing this double burden requires strategies beyond pharmacological therapy, with emphasis on lifestyle modifications such as healthy diets, physical activity, stress reduction, and community-based support [11]. Evidence from India and other low- and middle-income countries demonstrates that community-based interventions are effective in improving health outcomes [12]. Programs in Kerala have successfully reduced mean blood pressure and improved awareness and treatment adherence [13], while yoga-based and lifestyle education programs in India and Nepal have shown significant benefits in lowering blood pressure and preventing diabetes. These culturally tailored, low-cost approaches highlight the potential of community-driven interventions to complement clinical care in resource-limited settings [14]. Despite these promising findings, there remains limited evidence from randomized controlled trials (RCTs) that specifically address the combined management of diabetes and hypertension in the Indian context. Most studies have examined these conditions separately, leaving a critical gap in integrated approaches for individuals experiencing both simultaneously. This study seeks to address this gap by implementing and evaluating a structured community-based intervention designed to be implemented among individuals with coexisting diabetes and hypertension.

## Description of protocol

Diabetes and hypertension pose a major global public health challenge, with prevalence steadily increasing, particularly in low- and middle-income countries [12]. Managing these chronic conditions within community settings can be daunting, especially for individuals with limited health literacy, irregular follow-up care, and restricted access to healthcare services [11,12]. Poor treatment adherence, unhealthy lifestyle practices, and inadequate self-care behaviors are key factors contributing to uncontrolled blood pressure and blood sugar levels, ultimately leading to serious complications [16].

A community-based intervention (CBI) integrating yoga, health education, dietary counseling, and regular follow-up support has the potential to improve clinical outcomes and enhance the self-care capacity of patients with diabetes and hypertension. Structured yoga sessions may aid in regulating blood pressure and glucose levels, while targeted health education can strengthen patients’ knowledge and awareness regarding disease management. Dietary guidance supports better nutritional choices, and sustained follow-up encourages treatment adherence and lifestyle modification.

The significance of this trial lies in its potential to provide evidence for integrated chronic disease management, empower patients through improved health literacy, and promote cost-effective strategies that can be scaled within primary healthcare systems. Moreover, the outcomes of this study align with national and global priorities such as the WHO Global Action Plan and the Sustainable Development Goals, thereby contributing to the reduction of premature mortality from NCDs [15]. However, evidence regarding the effectiveness of such interventions remains inconclusive due to variability across studies in terms of patient characteristics, intervention intensity, duration of follow-up, and behavioral support strategies. Therefore, this study protocol outlines a structured and sustainable model of community-based intervention aimed at improving clinical outcomes (blood pressure and blood sugar control), quality of life, health literacy, and medication adherence among adults with diabetes and hypertension living in urban slum settings.

## Aim of the study

The primary aim of the study is to evaluate the effectiveness of a CBI that integrates yoga practices, health education, dietary counseling, regular follow-up, and structured monitoring in improving clinical outcomes (blood pressure and blood sugar control) and health literacy among individuals with coexisting diabetes and hypertension.

## Study design

This study will adopt an RCT design in accordance with the updated SPIRIT 2025 statement guidelines [17]. Eligible participants will be randomly assigned to either the intervention or control group.

## Participants and enrolment

The study will be conducted in selected urban slum communities under the Bhubaneswar Municipal Corporation (BMC), Odisha. The selection of participants will be based on population density and the presence of active Non-Communicable Disease (NCD). In collaboration with local health authorities, the slums will be chosen to ensure adequate representation and logistical feasibility for data collection and intervention delivery. A community health survey will be conducted by a trained researcher in coordination with Accredited Social Health Activists (ASHAs) and Auxiliary Nurse Midwives (ANMs). All data collectors will undergo a structured two-day training program focused on participant screening, ethical considerations, informed consent procedures, and standardized data collection protocols.

The training will be supervised by senior investigators to ensure uniformity and quality assurance. Periodic field monitoring and random checks will be conducted throughout the study to maintain data reliability and adherence to protocol. Eligible participants will include individuals aged between 30 and 60 years who have been previously diagnosed with both diabetes mellitus and hyrtension. Verification of the diagnosis will be carried out through multiple sources, including review of patients’ medical records, confirmation in the NCD registers and the Health Management Information System (HMIS), and validation by the CHOs or healthcare workers based on available clinical documentation. Self-reported cases without medical record confirmation will not be included. All eligible participants will receive a detailed explanation of the study’s purpose, procedures, and potential benefits and risks. Written informed consent will be obtained before enrolment. Following enrolment, participants will be followed up within their community settings throughout the intervention and data collection phases to ensure consistent participation and outcome monitoring.

## Inclusion and exclusion criteria

The inclusion criteria for this study will comprise adults aged between 30–60 years who have been diagnosed with both type 2 diabetes mellitus and hypertension for at least one year. Eligible participants must be residents of the selected community for at least the past six months and are expected to remain there for the next six months. In addition, participants should be receiving treatment from a healthcare provider and must be willing and able to provide informed consent for participation.

Exclusion criteria will include individuals diagnosed with only diabetes or only hypertension without the coexistence of both conditions. Patients with severe complications such as end-stage renal disease, stroke, or advanced cardiovascular disease, as well as those requiring hospitalization, will be excluded. Pregnant women, lactating mothers, and individuals with psychiatric illness, cognitive or physical impairments that limit participation, or those already enrolled in another interventional study will also be excluded. Furthermore, participants who are unwilling or unable to complete follow-up assessments will not be considered.

## Sample size

The sample size was calculated using the formula:

$$N=\frac{2\sigma^{2}{\left(Z_{1-\alpha /2}+Z_{1-\beta }\right)}^{2}}{\delta^{2}}$$

Where:

*N* = required sample size per group
σ² = pooled standard deviation of the primary outcome measure (assumed as 0.74 from prior studies)
δ² = Anticipated difference in mean outcomes between groups (0.40)
Z_1−α/2_ = standard normal variate corresponding to a 95 % confidence level (1.96)
Z_1−β_ = standard normal variate corresponding to 80 % power (0.84)

By applying these values, the estimated sample size is approximately 54 participants per group. Considering a 10 % attrition rate, the final sample size will be 120 participants (60 in the intervention group and 60 in the control group).

## Intervention group

Participants in the intervention group will receive a structured Community-Based Intervention (CBI) in addition to standard medical care. The CBI is designed to provide holistic care by integrating education, yoga-based lifestyle modification, and home-based monitoring. The intervention will be delivered in groups of 8–10 participants, led by the same trained nurse facilitator throughout the study, with support from a certified yoga instructor for yoga sessions. The complete intervention will span 6 weeks, with sessions conducted six days per week.

### Pre-Intervention orientation and baseline assessment

The program will begin with a pre-intervention orientation and baseline assessment conducted by the nurse facilitator in a community hall within the selected slum, chosen for its accessibility of participants’ homes. Family participation will be encouraged but is not mandatory. During orientation, participants will receive education on essential self-care strategies for managing diabetes and hypertension, including maintaining physical activity, adequate rest and sleep, fluid balance, dietary modifications, adherence to prescribed medications, stress reduction, and avoidance of lifestyle risk factors such as tobacco use and excess sodium intake. To individualize the intervention, the nurse will apply the Transtheoretical Model of Behaviour Change, which includes the five stages of pre-contemplation, contemplation, determination, action, and maintenance. This approach will guide tailored goal setting and help match the intervention to each participant’s motivational readiness for adopting healthier behaviors.

### Yoga-Based lifestyle modification and nurse-led coaching

Following orientation, participants will engage in daily yoga sessions of a minimum of 30–45 min, six days per week, conducted in a local community hall by a certified yoga instructor. The yoga program includes warm-up exercises, selected postures such as Sukhasana, Vajrasana, Trikonasana, Paschimottanasana, Pavanamuktasana, Bhujangasana, and Shavasana, as well as breathing practices like Kapalabhati and guided relaxation or meditation. These practices are intended to reduce stress, improve flexibility, and enhance cardiometabolic health. Complementing yoga practice, weekly nurse-led group coaching sessions will be organized to reinforce educational messages, address adherence challenges, clarify misconceptions, and foster peer support. Coaching will be adapted to the participant’s stage of behavior change, providing informational support in early stages and motivational reinforcement during the action and maintenance stages. To enhance sustainability, ASHAs will assist in motivating participants to continue the practice throughout the intervention and beyond. Sessions will be scheduled at convenient times for participants and held in community halls close to their residences to ensure accessibility.

### Home-Based monitoring and follow-up reinforcement

To strengthen continuity of care, participants will be trained in home-based monitoring of blood pressure and blood glucose and will maintain a lifestyle diary documenting daily yoga practice, diet, and medication adherence. The nurse will provide follow-up reinforcement through weekly contacts in the first month and biweekly thereafter, conducted via home visits or telephone calls. During these interactions, the nurse will review self-monitoring records, reinforce educational content, troubleshoot difficulties, and provide motivational support. Participants who show poor adherence or worsening clinical indicators will receive additional counseling and relate to peer support strategies to encourage engagement and compliance.

## Control arm

Participants randomized to the control group will continue to receive routine medical care as prescribed by their physician. This care will include regular consultations with a physician, prescribed medications, routine monitoring of blood pressure and blood glucose, and general guidance on diet, physical activity, and lifestyle management. The control group will not receive any additional interventions, such as educational sessions, yoga-based activities, or nurse-led counseling. This design provides a clear comparator for assessing the effectiveness of the structured community-based intervention delivered to the experimental group.

## Randomization process

A total of 120 participants will be randomly assigned to either the intervention or control groups. To ensure balance in group sizes throughout the trial, block randomization will be employed using 12 blocks, each containing 5 participants allocated to the intervention group and 5 to the control group. The randomization sequences for each block will be generated using a computer-based random number list. An independent researcher, who is not involved in the intervention delivery or data collection, will oversee the randomization process. This researcher will be responsible for generating the allocation sequence and preparing the allocation concealment materials but will not directly assign participants to groups. Allocation concealment will be maintained by placing each assignment in opaque, sequentially numbered, sealed envelopes. These envelopes will only be opened after a participant has been formally enrolled in the study, ensuring that group allocation remains unpredictable and free from selection bias.

## Data collection plan and management

The initial survey will be conducted within selected urban slum communities to identify individuals diagnosed with both diabetes mellitus and hypertension. Eligible participants will be approached at their households to ensure accessibility and convenience. Trained community health workers, under the supervision of the research team, will carry out the survey following standardized procedures to ensure consistency and accuracy. After screening for eligibility and obtaining written informed consent, baseline data will be collected using structured interviews. Once baseline assessments are completed, participants will be allocated to either the intervention or control group using a computer-generated block randomization procedure. Follow-up data will be collected at multiple time points: baseline, 2 months, 3 months, and 6 months to evaluate both short- and mid-term outcomes.

## Statistical analysis

Data analysis will be performed using SPSS version 28.0. Descriptive statistics, including mean, standard deviation, median, interquartile range, frequency, and percentage, will be calculated to summarize participant characteristics and outcome variables [18]. Inferential statistics, such as independent sample *t*-tests and repeated measures ANOVA, will be used to assess differences in outcomes within and between groups. The choice of parametric or non-parametric tests will depend on the distribution and normality of the data [19].

All analyses will follow the intention-to-treat principle, ensuring that each participant is analyzed in the group to which they were originally assigned, regardless of adherence to the intervention. Missing outcome data will be carefully addressed based on the nature of the missingness. For data missing at random, multiple imputation techniques will be applied. For data not missing at random, alternative approaches, such as last observation carried forward or mean imputation, will be employed. Effectiveness of the intervention will be expressed as differences in proportions with 95 % confidence intervals and 80 % statistical power. Comparisons will be made using appropriate statistical tests with adjustments for multiple comparisons

## Study outcomes

This study aims to evaluate the effect of a CBI strategy on clinical parameters, health literacy, medication adherence, and quality of life among individuals with both diabetes and hypertension. Data will be collected using structured and standardized instruments to ensure reliability and validity. Participants’ sociodemographic and clinical profiles will be recorded at baseline using a structured form to capture demographic details and health-related history.

### Clinical outcome

Clinical outcomes will include blood pressure and blood sugar levels. Blood pressure will be measured using a calibrated digital sphygmomanometer, with two readings taken and averaged to ensure accuracy. Blood glucose levels will be measured through random blood sugar test using a standardized glucometer. These measurements will be collected at baseline, 2 months, 3 months, and 6 months to track changes over time.

### Health literacy

Health literacy will be assessed using a modified Diabetes and Hypertension Health Literacy Scale, consisting of 14 items. This scale evaluates participants’ understanding of dietary habits, physical activity, and self-care practices for managing both conditions. The scale will be validated by 11 experts in the relevant fields, and reliability will be assessed with 10% of the total sample to ensure internal consistency. The scale was modified because existing instruments assessed diabetes and hypertension separately; the adapted version provides an integrated assessment suitable for participants with comorbid diabetes and hypertension. Health literacy assessments will be conducted at baseline, 2 months, 3 months, and 6 months.

### Medication adherence

Medication adherence will be measured using the standardized 8-item Morisky Medication Adherence Scale (MMAS-8), which evaluates various domains of medication-taking behavior in chronic disease management [20]. Assessments will occur at baseline, 2 months, 3 months, and 6 months, providing repeated measures to track adherence over time.

### Quality of life

**Fig. 1:**
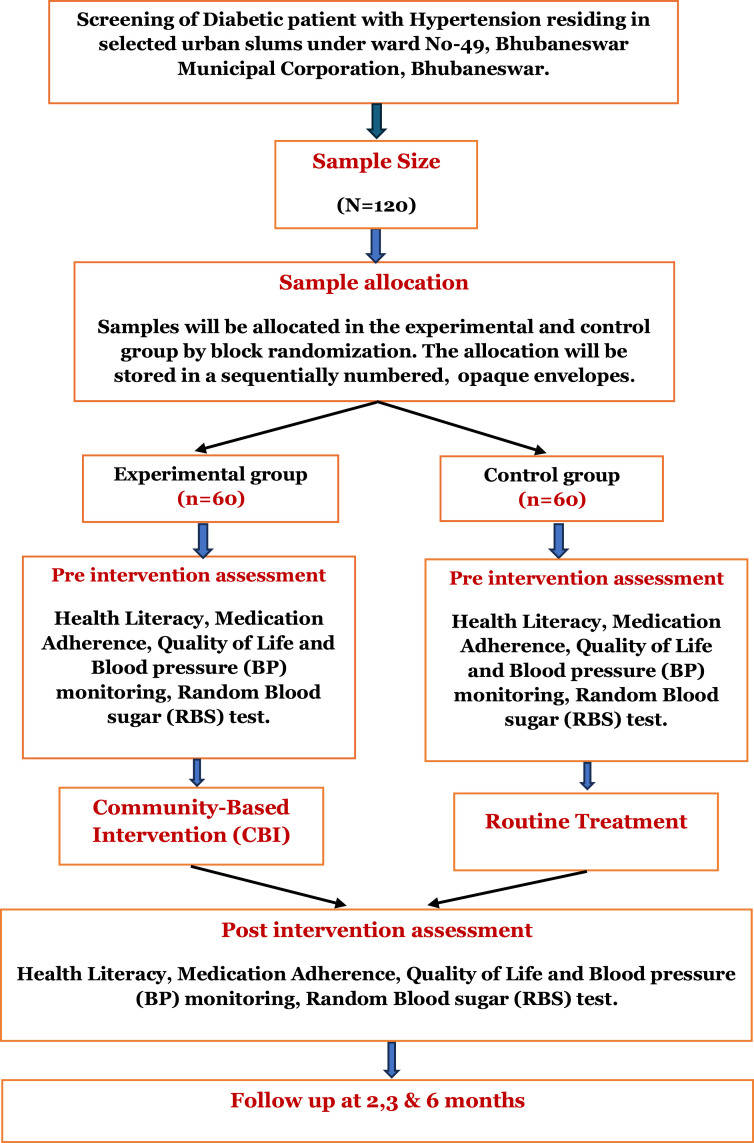
Participant timeline across various time points.

Quality of life will be assessed using a modified questionnaire derived from the Diabetes Quality of Life Questionnaire (DQOL) and the High Blood Pressure Quality of Life Questionnaire (HBP-QLQ), consisting of 16 items covering physical, psychological, social, and environmental domains. The scale will be validated by 11 experts, and reliability will be assessed with 10 % of the sample to ensure internal consistency. Similar to the health literacy scale, this instrument was modified to integrate assessment of both diabetes and hypertension for accurate evaluation in participants with comorbid conditions. Assessments will be conducted at baseline, 2 months, 3 months, and 6 months. The complete participant assessment timeline is illustrated in the CONSORT diagram (Fig. 1).

### Follow-Up and data collection procedures

Follow-up questionnaires and clinical measurements will be administered through in-person home visits to ensure convenience and maximize participation. Participants will complete self-report at their homes, guided by trained research staff to ensure accurate and complete responses. Clinical measurements, including blood pressure and blood glucose monitoring, will also be conducted during home visits. To promote adherence to scheduled assessments and minimize loss to follow-up, participants will receive prior reminders through telephone calls and text messages. The researcher, with support from local ASHA workers, will maintain regular contact with participants to reinforce the importance of completing each assessment and provide support for any challenges. Flexible scheduling of visits, tailored to participants’ availability, will further enhance compliance. Home visits will be conducted for participants unable to attend community sessions, ensuring continuity of data collection and minimizing attrition.

## Ethical considerations

The study received approval from the Institutional Ethics Committee (IEC) of the Institution (Reference No: IEC/IMS.SH/SOA/2022/433). The trial has been prospectively registered in the Clinical Trial Registry of India (CTRI/2024/08/071,775).

Informed consent will be obtained from all participants before enrolment. The consent form will be provided in both English and Odia to ensure participants fully understand the study procedures. To maintain confidentiality, personal information will not be shared with third parties. Data confidentiality and anonymity will be maintained strictly throughout all phases of the study, data collection, analysis, reporting, and publication.

## Protocol validation

The rising prevalence of coexisting diabetes and hypertension, particularly in underserved urban communities, presents significant challenges for effective disease management and long-term health outcomes [21]. Conventional care often focuses on pharmacological treatment alone, neglecting the role of lifestyle modification, patient education, and self-management support, which are critical for achieving optimal glycemic and blood pressure control [22,23]. Evidence suggests that structured community-based interventions, including health education, yoga-based lifestyle modification, dietary counseling, and regular monitoring, can improve treatment adherence, enhance health literacy, and positively influence quality of life [24,25]. However, there is limited rigorous evidence from randomized controlled trials in urban slum populations, where barriers such as low health literacy, limited access to care, and socioeconomic constraints may impede successful management [26,27]. Conducting this RCT will allow for a systematic evaluation of the effectiveness of a comprehensive CBI strategy compared to standard care. The study’s design, including block randomization, standardized outcome assessment, and follow-up at multiple time points, ensures methodological rigor and minimizes bias. Findings from this trial are expected to provide evidence-based guidance for implementing scalable, patient-centered interventions that integrate lifestyle, education, and clinical care, thereby addressing the gap in chronic disease management within vulnerable populations.

To the best of our knowledge, this protocol is novel and has not been implemented in any prior settings; therefore, no preliminary data are currently available.

## Limitations

This randomized controlled trial has several limitations. First, it is conducted in selected urban slums, which may limit generalizability to rural areas or populations with higher socioeconomic status. Participants’ sociodemographic profiles will be documented to provide contextual interpretation of the findings. Second, blinding is not employed, and participants are aware of their group allocation, which may introduce performance bias, particularly in self-reported outcomes. To mitigate this, validated assessment tools will be used, and data will be collected by trained personnel who are not involved in the intervention. Third, the intervention relies on participants’ attendance at yoga and counseling sessions, as well as adherence to home-based monitoring. Variations in participation may influence outcomes; therefore, reminder calls, motivational support, and flexible scheduling will be implemented to enhance adherence. Fourth, potential confounding factors such as concurrent treatments, dietary changes, or lifestyle modifications cannot be fully controlled. Baseline assessments and ongoing monitoring will help account for these factors during analysis.

Additionally, given the study’s focus on urban slum populations, access to regular healthcare and diagnostic services may be limited. This could lead to underdiagnosis or delayed identification of chronic conditions, affecting the representativeness of the recruited sample. Consequently, the findings are most generalizable to similar urban, resource-constrained communities rather than broader urban or rural populations. Nevertheless, by leveraging existing public health infrastructure, including urban primary health centers, NCD registers, and frontline health workers, the study reflects real-world conditions and aims to generate contextually relevant evidence that can inform scalable community-based interventions in comparable low-resource settings.

## Funding support

None

## CRediT authorship contribution statement

**Krishna Kumari Samantaray:** Conceptualization, Methodology, Writing – original draft. **Sasmita Das:** Conceptualization, Methodology, Writing – review & editing. **Krushna Chandra Sahoo:** Conceptualization, Methodology, Writing – review & editing. **N Siva:** Methodology, Supervision, Validation, Writing – review & editing.

## Declaration of competing interest

The authors declare that they have no known competing financial interests or personal relationships that could have appeared to influence the work reported in this paper.

## Data Availability

No data was used for the research described in the article.
